# The myxozoans *Myxobolus cerebralis* and *Tetracapsuloides bryosalmonae* modulate rainbow trout immune responses: quantitative shotgun proteomics at the portals of entry after single and co-infections

**DOI:** 10.3389/fcimb.2024.1369615

**Published:** 2024-05-13

**Authors:** Mona Saleh, Karin Hummel, Sarah Schlosser, Ebrahim Razzazi-Fazeli, Jerri L. Bartholomew, Astrid Holzer, Christopher J. Secombes, Mansour El-Matbouli

**Affiliations:** ^1^ Division of Fish Health, University of Veterinary Medicine, Vienna, Austria; ^2^ VetCore, University of Veterinary Medicine, Vienna, Austria; ^3^ Department of Microbiology, Oregon State University, Corvallis, OR, United States; ^4^ Scottish Fish Immunology Research Centre, School of Biological Sciences, University of Aberdeen, Scotland, United Kingdom

**Keywords:** salmonids, proteome, immunomodulation, proliferative kidney disease, whirling disease

## Abstract

**Introduction:**

Little is known about the proteomic changes at the portals of entry in rainbow trout after infection with the myxozoan parasites, *Myxobolus cerebralis*, and *Tetracapsuloides bryosalmonae*. Whirling disease (WD) is a severe disease of salmonids, caused by the myxosporean *M. cerebralis*, while, proliferative kidney disease (PKD) is caused by *T. bryosalmonae*, which instead belongs to the class Malacosporea. Climate change is providing more suitable conditions for myxozoan parasites lifecycle, posing a high risk to salmonid aquaculture and contributing to the decline of wild trout populations in North America and Europe. Therefore, the aim of this study was to provide the first proteomic profiles of the host in the search for evasion strategies during single and coinfection with *M. cerebralis* and *T. bryosalmonae*.

**Methods:**

One group of fish was initially infected with *M. cerebralis* and another group with *T. bryosalmonae*. After 30 days, half of the fish in each group were co-infected with the other parasite. Using a quantitative proteomic approach, we investigated proteomic changes in the caudal fins and gills of rainbow trout before and after co-infection.

**Results:**

In the caudal fins, 16 proteins were differentially regulated post exposure to *M. cerebralis*, whereas 27 proteins were differentially modulated in the gills of the infected rainbow trout post exposure to *T. bryosalmonae*. After co-infection, 4 proteins involved in parasite recognition and the regulation of host immune responses were differentially modulated between the groups in the caudal fin. In the gills, 11 proteins involved in parasite recognition and host immunity, including 4 myxozoan proteins predicted to be virulence factors, were differentially modulated.

**Discussion:**

The results of this study increase our knowledge on rainbow trout co-infections by myxozoan parasites and rainbow trout immune responses against myxozoans at the portals of entry, supporting a better understanding of these host-parasite interactions.

## 1 Introduction

Whirling disease (WD) is a severe disease of salmonids, caused by the myxozoan parasite *Myxobolus cerebralis* (Hofer, 1903; Hoffman, 1990). *M. cerebralis* has a complex life cycle that alternates between two hosts, the invertebrate oligochaetae *Tubifex tubifex* and a vertebrate salmonid host (Wolf and Markiw, 1984). Climate change is providing more suitable conditions for myxozoan parasites lifecycle, posing a high risk to salmonid aquaculture and contributing to the decline of wild trout populations in North America and Europe (Hedrick et al., 1998; Schisler et al., 2000; Wahli et al., 2008; Akram et al., 2023). A genome-wide expression profiling study found several immune related genes to be significantly up-regulated in the skin of resistant and susceptible rainbow trout following exposure to *M. cerebralis* (Baerwald et al., 2008). Most of the annotated genes with known molecular functions were involved in the IFNγ signalling pathway. Several gene expression studies have also explored the mechanisms of host resistance to WD (Rucker and El-Matbouli, 2007; Severin and El-Matbouli, 2007; Severin et al., 2010; Baerwald, 2013; Saleh et al., 2020a; 2020b). The results suggest that susceptible rainbow trout is unable to mount an efficient immune response upon the infection with *M. cerebralis*. Indeed, *M. cerebralis* induces early cellular responses at the infection sites resulting in excessive local inflammatory responses, which likely enhance tissue damage caused by the parasite and support *M. cerebralis* immune evasion and replication (Saleh et al., 2019a). Conversely, effective local and systemic immune reactions and appropriate stimulation of T lymphocytes, such as CD4^+^ T helper cells are essential for host protection during *M. cerebralis* infection. Indeed, one major evasion strategy of *M. cerebralis* appears to involve modulation of Th17 immunity through the STAT3/SOCS-3/IL-6 axis by inducing SOCS-3 and influencing the balance between Treg cells and pro-inflammatory Th17 cells (Saleh et al., 2020a). A balanced Th17/Treg response is key to induce protective immunity and contributes to WD resistance.

Proliferative kidney disease (PKD) is caused by another myxozoan, *Tetracapsuloides bryosalmonae*, which instead belongs to the class Malacosporea (Canning et al., 2000). *T. bryosalmonae* alternates between an invertebrate host, the freshwater bryozoan *Fredericella sultana*, and a vertebrate salmonid host (Morris and Adams, 2006; Grabner and El-Matbouli, 2010). The disease targets the kidney and induces a chronic immunopathology, granulomatous-like lesions and lymphocytic hyperplasia of the interstitial kidney parenchyma, with hyperimmunoglobulaemia (Gorgoglione et al., 2013). PKD is widespread in Europe and North America and causes great losses to farmed and wild salmonids (Gorgoglione et al., 2016a; Bailey et al., 2021). PKD pathogenesis is temperature-dependent, and is spreading as a result of climate change (Okamura et al., 2015). Infective *T. bryosalmonae* malacospores are dispersed by infected tolerant hosts, such as brown trout (*Salmo trutta*) in Europe and by infected bryozoan distribution, inducing through statoblasts dispersion and migration of infected zooids (Abd-Elfattah et al., 2014; Gorgoglione et al., 2016b; Abd-Elfattah et al., 2017). Fish mortality varies from ≤20% up to 95–100% in severe outbreaks associated with secondary infections and unfavorable farming or environmental conditions (Okamura et al., 2011; Gorgoglione et al., 2016b). Fish that survive PKD may acquire a strong immunity and may develop resistance to re-infection (Gorgoglione et al., 2013; Bailey et al., 2020).

Several transcriptional studies have provided key knowledge on the mechanisms involved in fish disease resistance against myxozoan parasites (Rucker & El-Matbouli 2007; Severin & El-Matbouli 2007; Baerwald et al., 2008; Severin et al., 2010; Baerwald, 2013; Sitjà-Bobadilla et al., 2015; Saleh 2019a; 2020a; Holzer et al., 2021). However, further study is required to understand comprehensively the immune response of fish during myxozoan infections, including during co-infections. The interaction between myxozoans in the host can be synergistic or antagonistic. Environmental changes influence the interactions of myxozoans and may cause outbreaks of fish diseases (Okamura et al., 2015). Indeed, temperature influences the kinetics of *T. bryosalmonae* development in the host and similar events might occur for other myxozoan species (Gay et al., 2001). Mixed infections with five myxozoan species (*T. bryosalmonae*, *Sphaerospora truttae*, *Chloromyxum schurovi*, *Chloromyxum truttae* and *Myxobolus* species) were observed in brown trout, in samples collected from farms in central Scotland (Holzer et al., 2006). Natural co-infections with myxospores of a *Myxobolus* sp. and *Henneguya* sp. were also reported in the posterior kidney of the pond-reared fish *Piaractus mesopotamicus* from Southeast Brazil (Manrique et al., 2017). A recent study reported that the highly diverse group of Neotropical fishes are hosts of a multitude of myxozoan parasites including *Myxobolus* species and that they represent an interesting research area for conservation and economic reasons in Mexico (Alama-Bermejo et al., 2023).

During co-infections the host immune response induced by one pathogen modulates the pathogenesis of subsequent infections by suppression or stimulation of the immune system. The transcriptional modulation of the immune response of rainbow trout has been assessed during co-infection in the posterior kidney and cranial cartilages, the main tissues targeted during PKD or WD pathogenesis (Kotob et al., 2018). The results highlighted the role of the SOCS/JAK/STAT signaling pathway during co-infection with the two myxozoan parasites. The impact of co-infection with *T. bryosalmonae* and *M. cerebralis* on the pathology of rainbow trout was also examined (Kotob et al., 2017).

While we have a general overview of the cellular basis of the immune response of rainbow trout to these two myxozoans, the proteomic changes underlying the immune responses of fish to myxozoan parasites remain unclear. Hence, the aim of this study was to undertake the first proteomic profiling of infected fish, at the portals of entry, to explore potential host evasion strategies of *M. cerebralis* and *T. bryosalmonae*. We collected the caudal fin, as it has been reported to be significantly more attractive to *M. cerebralis* than gills or skin by showing the parasite highest intensity and confirming its suitability for detecting early stages of the parasite (Skirpstunas et al., 2006; Eszterbauer et al., 2019). Furthermore, in our previous studies, caudal fin has enabled us to monitor the local cellular and cytokine immune responses in rainbow trout (Saleh et al., 2019a; 2020a). The gill was identified as a portal of entry for *T. bryosalmonae* and attached/penetrating stages were found only on or in the gills, and not in the skin (Morris et al., 2000; Longshaw et al., 2002; Grabner and El-Matbouli, 2010). The proteins were analysed specifically to provide information on host immune responses, host-parasite interactions and biological processes, and pathways activated by the co-infections. The proteins identified may have value as markers for the diagnosis of fish disease and to develop novel approaches for disease management.

## 2 Methods

### 2.1 Experiment design

Pathogen-free rainbow trout (90 days-old, mean length 4.02 ± 0.26 cm, mean weight 0.6 ± 0.15 g) were distributed in tanks (*n* = 3) receiving 15°C water and were fed (1% BW/day) daily with floating trout pellets (Aqua Garant, Austria). The North American strain (TL) rainbow trout with known susceptibility to both myxozoans was used for the exposure experiment (Rucker and El-Matbouli, 2007; El-Matbouli et al., 2009). Prior to the exposure trail, fish (*n* = 10) were randomly sampled and tested for *M. cerebralis* and *T. bryosalmonae* by qPCR (Kotob et al., 2018). The fish were observed for signs of morbidity (uncoordinated swimming and/or lethargy) three times a day for timely removal and euthanizing of such fish where needed.

**Figure 1:**
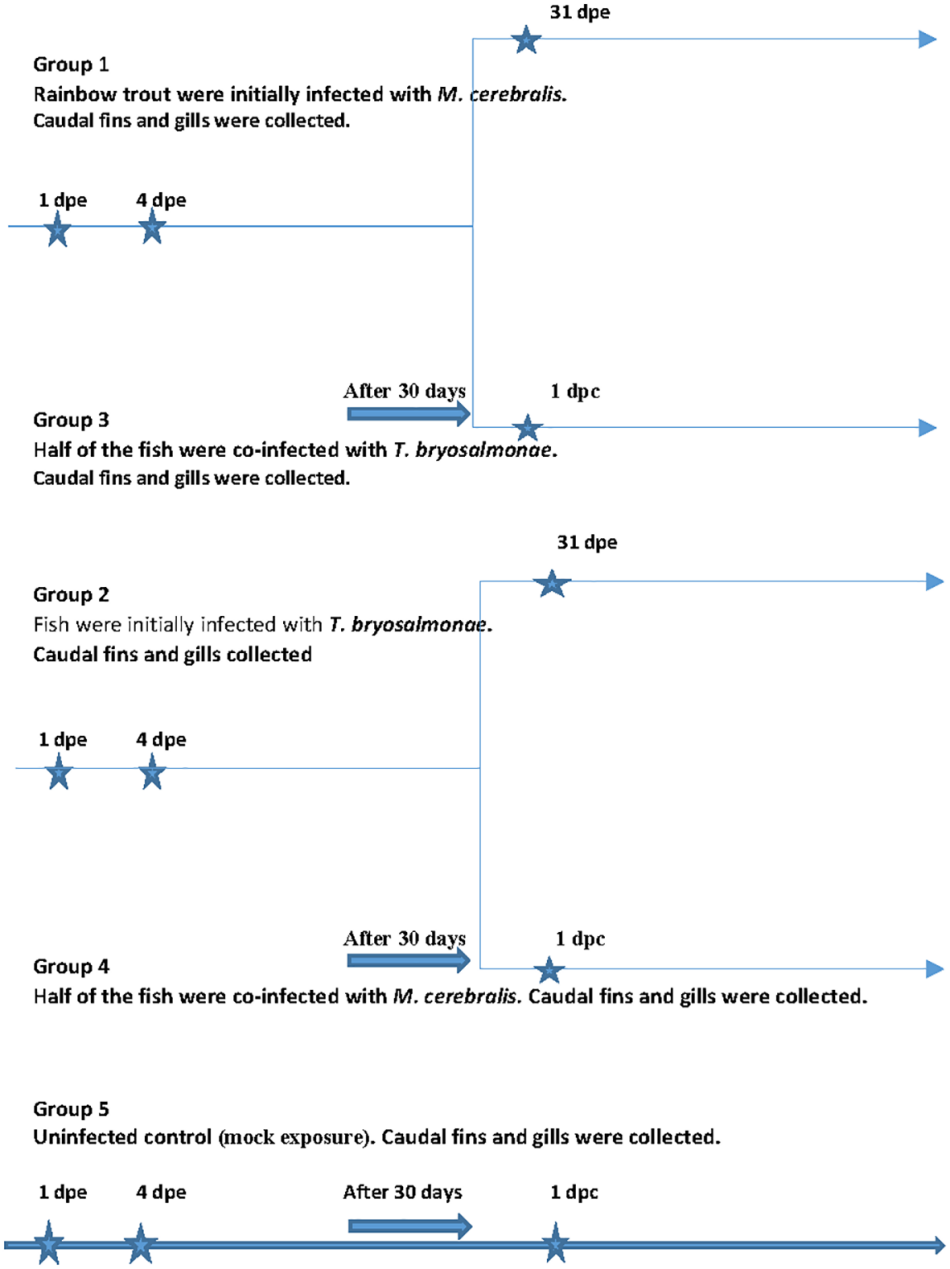
*In vivo* experimental outline. The scheme shows the experimental design otrial. A shows the sampling time points.

The fish were initially divided into three groups (**Figure 1**). The first group (group 1) fish (*n* = 36) were exposed to *M. cerebralis* triactinomyxons (TAMs) (2000 TAMs/fish), while fish (*n* = 36) in the second group (group 2) were exposed to free *T. bryosalmonae* spores released from 22 mature parasite sacs at 16°C (Kotob et al., 2017). The TAMs and the spores were produced as described in Saleh et al. (2019a) and Kotob et al. (2017). Thirty days after infection, half of the fish from group 1 and group 2 (single infection groups) were reciprocally co-infected with the other parasite, separately [group 3 (*n* = 18) and group 4 (*n* = 18)]. The last group (group 5) fish (*n* = 27) were subjected to mock exposure to specific pathogen free water and used as a negative control. At 1 day and 4 day as well as 31 days post exposure (dpe) (day 1 post co-infection (dpc)), fish (*n* = 9) from each group were euthanized and sampled. Caudal fins (portal of entry of *M. cerebralis*) and gills (portal of entry of *T. bryosalmonae*) were washed with sterile phosphate buffer and stored at -80°C until further analysis. Before, at zero time point and at all time points during the experiment, fish (*n* = 9) from each group were screened for parasites by microscopic examination of gill and skin scrapings and kidney swabs were taken to assess for bacterial agents by culture on blood agar plates as previously described (Anacker & Ordal, 1959). No infectious agents were detected prior to or during the experiment. In addition, before and at all time points during the experiment, homogenates of brain, entire viscera, spleen and kidney were subjected to viral screenings using BF-2 and EPC cell lines according to standard cell culture methods (Manual of Diagnostic Tests for Aquatic Animals, 2021). No virus was detected before or throughout the experiment. During the *in vivo* experiments, the parasite load of *M. cerebralis* and *T. bryosalmonae* was confirmed by qPCR at all-time points as previously described (Kotob et al., 2018).

### 2.2 RNA isolation and cDNA synthesis

Total RNA was extracted from the caudal fin and gill samples at each time point using RNeasy Mini Kit (Qiagen, Hilden, Germany) according to the manufacturer’s instructions. To remove any residual DNA contamination, an on-column DNase digestion step was performed. RNA concentration and quality control were determined using a Nanodrop 2000c spectrophotometer and by agarose gel electrophoresis (Thermo Fisher Scientific, Wilmington, USA). The isolated RNA was used to synthesize cDNA, utilizing iScript cDNA Synthesis Kit (Bio-Rad, Munich, Germany) according to the manufacturer’s instructions. The synthesized cDNA was diluted with nuclease-free water, aliquoted and stored at −20°C until further analysis.

### 2.3 Quantitative real-time PCR

Parasite load determination for *M. cerebralis* (using the forward primer Myx18-909 F CTTTGACTGAATGTTATTCAGTTACAGCA and the reverse primer Myx18-996 R GCGGTCTGGGCAAATGC) and *T. bryosalmonae* (using the forward primer RPL18 F GTAAACGGGGACAAAAAGA and the reverse primer RPL18 R GGAGCAGCACCAAAATAC), was performed using samples from caudal fin and gills as described previously (Kelley et al, 2004; Gorgoglione et al., 2013). The PCR reaction of 20 μl final volume contained 4 μl of 1:10-fold diluted cDNA, 1× SsoAdvanced Universal SYBR Green Supermix (Bio-Rad), 0.4 μM of each primer, and DEPC-treated sterile distilled water (Bio-Rad). The PCR reaction consisted of an initial 5 min of cDNA denaturation at 95°C, followed by 35 cycles of 95°C for 30 s, 57–62°C for 30 s and 72°C for 30 s. The detection thresholds for Myx18 and RPL18 were 36.43 and 37.61, respectively. A melting-point curve was measured, starting from 55°C with an increase of 0.5°C at every 10 s up to 95°C, for detecting non-specific binding. The quantity of gene expression level was assessed using CFX96 Touch Real-Time PCR detection system (Bio-Rad, Munich, Germay). Elongation factor 1 alpha was used as a reference gene for normalizing the expression of targeted genes (Kumar et al., 2014). Calculation of relative gene expression was performed using CFX manager software version 3.1 (Bio-Rad, Munich, Germany).

### 2.4 Protein extraction

Control and infected fish tissue samples were processed for proteomic analysis. Caudal fin and gill tissues were solubilized using 400 µl pre-cooled denaturing lysis buffer (7M urea, 2M thiourea, 4% CHAPS and 1% DTT) containing mammalian protease inhibitor cocktail (Sigma Aldrich, Vienna, Austria). Sample suspensions were disrupted by sonication. The lysates were incubated overnight at 4°C, vortexed and centrifuged at 18000 x g for 30 min at 4°C and the supernatants collected. The total protein concentration of each lysate was determined colorimetrically with a NanoDrop 2000c spectrophotometer and using the Pierce 660 nm Protein Assay according to the manufacturer’s instructions (Thermo Scientific, Vienna, Austria).

### 2.5 Protein digestion and nanoLC-ESI-Orbitrap-MS/MS analysis

Sample preparation was performed using a Filter-Aided-Sample preparation-Protocol (FASP) according to Wiśniewski et al. (2009) and Wiśniewski et al. (2016) as described in Ramires et al. (2022). In brief, 30 µg of protein were digested on a 10 kDa Pall Nanosep centrifugal device (Cytiva, MA, USA).

After reduction with dithiothreitol and alkylation with iodoacetamide, proteins were digested using Trypsin-LysC-Mix from Promega (WI, USA). In order to stop the trypsin digestion, 5% trifluoroacetic acid was added. Peptides were extracted in three rounds each of 50 µL 50 mM Tris followed by centrifugation. Peptides were acidified using trifluoroacetic acid to a pH below 2. After C18 clean-up with spin columns (Pierce, Thermo Fisher), peptides were separated on a nanoRSLC system equipped with a 25 cm Acclaim PepMap C18 column (Thermo Fisher) and analysed on a high-resolution Q Exactive HF Orbitrap mass spectrometer as described in Gutiérrez et al. (2019).

### 2.6 Data processing and quantification

The database search was performed using the Proteome Discoverer Software 2.4.1.15 (Thermo Fisher Scientific). The protein databases were downloaded from the UniProt homepage (http://www.uniprot.org) for rainbow trout (taxonomy ID 8022), and myxozoa (taxonomy ID 35581). Search settings were as follows: 10 ppm precursor mass tolerance and 0.02 Da fragment mass tolerance; dynamic modifications allowed were oxidation of methionine as well as the N-terminal protein modifications acetylation, methionine loss and the combination of both, and static modification of carbamidomethylation on cysteine. For protein identification, only proteins with at least two identified peptides were reported. Intensity-based label free quantification was used in order to compare protein abundance in the experiments. Protein abundance was extracted from mass spec raw data in Proteome Discoverer software followed by normalization to total area sums. Exported normalized abundance values were filtered in Excel to remove proteins with missing values before subsequent statistical analysis using R programming language according to R Core Team (2021).

### 2.7 Bioinformatic analysis of the differentially regulated proteins

To determine differentially expressed proteins in the trout tissue samples, statistical evaluation was performed in the R programming language. The differential expression of proteins was evaluated using one-way analysis of variance (ANOVA) for each protein. To compensate for multiple testing, the method of Benjamini and Hochberg (1995) was used to control the false discovery rate (FDR). The differences were considered significant if FDR-adjusted *p* values were smaller than the significance level of *α* = 0.01. For such proteins, the honest significant difference method of Tukey was applied as a *post hoc* test to assess the significance of the pairwise comparisons. Protein expression was considered differential if the adjusted *p*-value was below *α* and the absolute fold change at least two (fold change <−2 or >+2). The mass spectrometry proteomics data have been deposited to the ProteomeXchange (Deutsch et al., 2023) Consortium via the PRIDE (Perez-Riverol et al., 2022) partner repository (Perez-Riverol et al., 2016) with the dataset identifier PXD050412. The biological processes, cellular components and molecular functions of the modulated proteins were assigned. Kyoto Encyclopedia of Genes and Genomes (KEGG) PATHWAY database was employed to predict involved pathways. To determine the protein-protein functional interaction network of the differentially regulated proteins, amino acid sequences were assessed using STRING v11.0 (Szklarczyk et al., 2017). The representation of the protein-protein network was analysed in the database, experiment, text mining, co-expression, neighborhood, gene fusion and co-occurrence.

## 3 Results

### 3.1 Myxobolus cerebralis and Tetracapsuloides bryosalmonae burden

**Figure 2:**
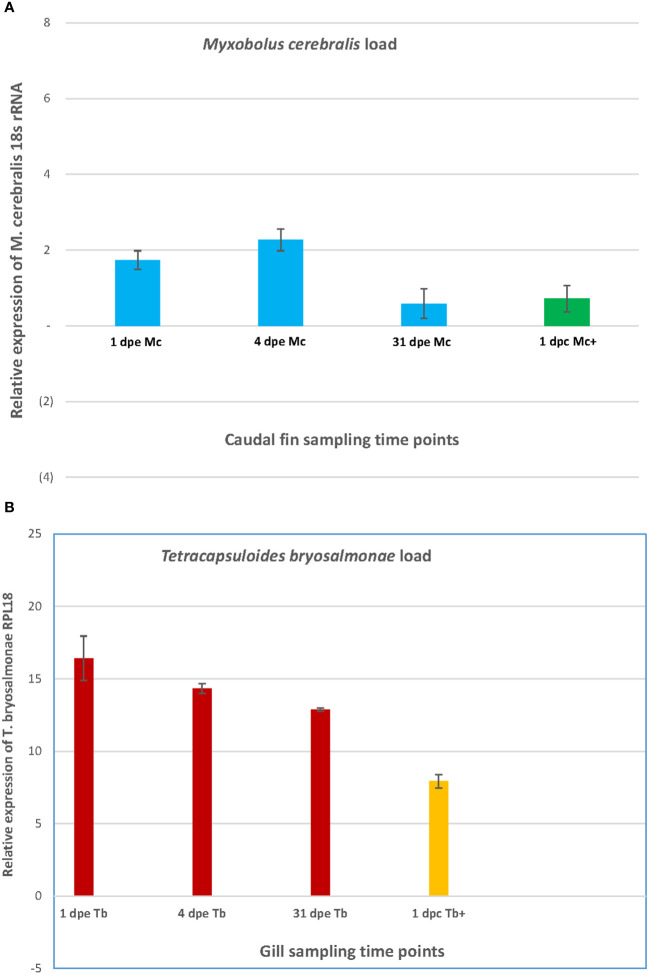
The graph shows the parasite burden of *M. cerebralis* **(A)** and *T. bryosalmonae* **(B)** in caudal fin and gills.

In the caudal fin and the gill tissues, the relative expression of the 18S rRNA of *M. cerebralis* and the 60S ribosomal protein L18 of *T. bryosalmonae* (RPL18) genes is shown in single and co-infections (**Figure 2**). The highest load of *M. cerebralis* was observed in the Mc group on day 4. The lowest parasite count was observed in the Mc group, while the Mc+ group had a higher load than the Mc on day 31 (1 dpc) (**Figure 2A**).

The highest load of *T. bryosalmonae* was observed in the Tb group on day 1. The *T. bryosalmonae* load decreased over time and the lowest parasite count was observed in the Tb+ group on day 31 (1 dpc) (**Figure 2B**).

### 3.2 Proteins differentially regulated in caudal fins after single infection with *M. cerebralis*

The proteomic analysis of caudal fin tissues showed 16 different proteins functioning in signal transduction, immune protection, metabolism and tissue repair were significantly modulated after 1 and 4 dpe to *M. cerebralis* (**Table 1**).

**Table 1:** Differentially regulated proteins in caudal fins post *M. cerebralis* infection; C, Control; D, Day; Mc *M. cerebralis* single infection; Asterisks show significant fold changes; 100 was used for fold changes of 100 or more.

Accession	Protein	McD1/CD1	McD4/CD1	McD4/CD4	McD4/McD1
A0A8C7RY94	Ly6/uPAR domain-containing protein	3.1	12.3*	11.2*	3.9*
A0A8C7VZU2	SH3_10 domain-containing	-1.0	-100*	-100*	-100*
A0A8C7RGF9	Clathrin heavy chain	-1.0	-1.0	100*	1.0
A0A8C7TSH9	Interferon-induced GTP-binding protein Mx	2.0	4.1*	4.9*	2.0
A0A8C7TPP7	Interferon-induced GTP-binding protein Mx	100*	100*	100*	1.4
A0A8C7PSW3	Fibulin-1	100*	100*	1.1	1.0
A0A060W7Y5	Laminin EGF-like domain-containing protein	100*	100*	-1.1	1.1
A0A8C7T444	PARP catalytic domain-containing protein	1.5	5*	4.5*	3.3*
A0A060YY05	PARP catalytic domain-containing protein	3.1	8.8*	4.7*	2.9
A0A060ZHX0	Slingshot protein phosphatase 1a	37*	47.4*	8.8	1.3
A0A8K9UZE1	Derlin/Dislocation and degradation of misfolded proteins	100*	100*	100*	-1.1
A0A8C7R7N2	TLDc domain-containing protein	1.0	100*	100*	100*
A0A8K9UT82	Cytidine monophosphate (UMP-CMP) kinase 2	5.5	23.7*	24.3*	4.3
A0A8C7TEV4	Succinyl-CoA:3-ketoacid-coenzyme A transferase (SCOT)	1,0	100*	-1,0	100*
A0A8K9WTU0	Barrier-to-autointegration factor (BAF)	2.1	7.4*	3.4	3.6
A0A8K9WZH0	Eukaryotic translation initiation factor 2B (EIF2B)	-1.1	1.1	100*	1.2

Among the differentially regulated proteins in caudal fins, a Ly6 (lymphocyte antigen-6)/uPAR (urokinase-type plasminogen activator receptor) superfamily member was significantly upregulated following exposure to *M. cerebralis*. Additionally, an SH3-domain-containing protein was extremely reduced at 4 dpe to *M. cerebralis*.

Clathrin heavy chain protein, a further differentially regulated protein identified in caudal fins, was on the other hand significantly induced at 4 dpe to *M. cerebralis*.

Of importance, two interferon IFN induced GTP-binding Mx proteins with functions in host immunity and leucocyte activation showed elevated protein abundance, increasing with time from day 1 to day 4 followed by proteins involved in tissue repair at day 4.

Rainbow trout fibulin-1 was highly upregulated in the current investigation at day 1 and day 4 after exposure to *M. cerebralis* as well as at day 4 in control fish.

The abundance of the laminin EGF-like domain-containing protein was also highly increased in the caudal fin tissues at day 1 and day 4 after exposure to *M. cerebralis*, as well as at day 4 in control fish.

In this proteome analysis, the abundance of two poly polymerase (PARP) catalytic domain-containing proteins were induced at 4 dpe to *M. cerebralis*.

Further, in this analysis, the abundance of a slingshot-1 protein was significantly induced at 1 and 4 dpe to *M. cerebralis*. The slingshot-1 protein showed higher abundance at 4 than at 1 dpe, as compared to the control group. Furthermore, the level of derlin was highly increased at 1 and 4 dpe to *M. cerebralis* as compared to the control group.

In the current study, the abundance of the anti-oxidative and anti-inflammatory TLDc domain-containing protein was highly induced in the caudal fin tissues at 4 dpe to *M. cerebralis* as compared to 1 dpe infected fish and control.

In this investigation, the abundance of cytidine monophosphate kinase 2 (CMPK2) was induced in rainbow trout caudal fin tissues at 1 dpe and showed significant upregulation at day 4 as compared to control and 1 dpe to *M. cerebralis*.

Following exposure to *M. cerebralis*, the abundance of succinyl-CoA:3-ketoacid-coenzyme A transferase (SCOT) was elevated at day 4 in this study.

Another protein significantly increased, at day 4, in caudal fins after *M. cerebralis* exposure was the barrier to autointegration factor (BAF). Similarly, the eukaryotic translation initiation factor 2B (EIF2B) was increased at day 4 in infected rainbow trout following exposure to *M. cerebralis*.

**Figure 3:**
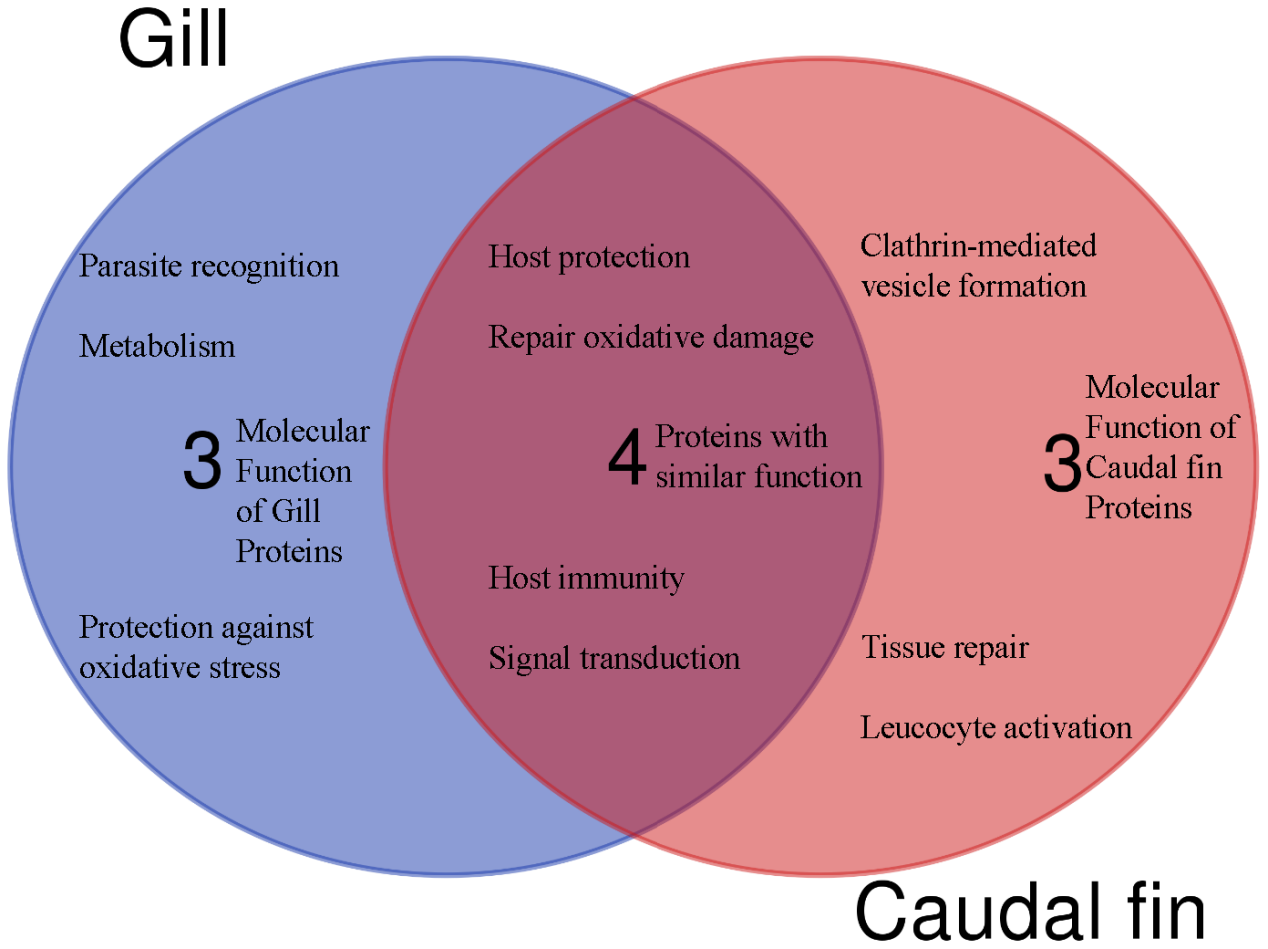
The Venn diagram shows the main molecular function and the number of unique or shared differentially regulated proteins in the caudal fin and gill, based on annotation and protein domain characterization. Venn diagram was designed using the web tool: https://bioinformatics.psb.ugent.be/webtools/Venn/.

Based on protein domain characterization of the annotated proteins that were significantly modulated in the caudal fin tissues, various molecular functions appear to be regulated, including leucocyte activation, host protection and tissue repair (**Figure 3**).

### 3.3 Proteins differentially regulated in caudal fins after co-infection

After 31 days of single-infection and 1 day of co-infection, caudal fin tissues had 4 proteins that were differentially regulated between the groups (**Table 2**). The complement factor H protein showed the highest abundance in the co-infection groups compared to single infection groups.

**Table 2:** Differentially regulated proteins of the caudal fin 31 dpe and 1 dpc; C, Control; D, Day; Mc and Mc+, *M. cerebralis* single and co-infection, Tb and Tb+, *T. bryosalmonae* single and co-infection; Asterisk shows significant fold change; 100 was used for fold changes of 100 or more.

Accession	Protein	Mc+/C	Tb+/C	Mc+/Mc	Tb+/Mc	Tb+/Tb	Tb+/Mc+
A0A8C7RPE6	Complement factor H-like	1.5	-1.2	100*	100*	100*	-1.8
A0A060XWK1	TPR_REGION domain-containing protein	-1.0	-100*	1,2	-100*	-100*	-100*
A0A8C7RXA9	Serine/threonine protein phosphatase 2A regulatory subunit	-6.1*	-4.6*	-4.2*	-3.2	-3.5	1.3
A0A8K9WUA7	Secretion associated Ras related GTPase 1B	-100*	-1.8	1.0	100*	100*	100*

The ras related GTPase protein, which is involved in parasite recognition, showed highest abundance in Tb+ groups (initially infected with *T. bryosalmonae* then after 30 days subsequently infected with *M. cerebralis*). On the other hand, a decreased abundance (specifically in Mc+ fish) was observed for a serine/threonine protein phosphatase, which has a role in the regulation of the stress response, perhaps indicating the failure of coping with stress caused by the parasites. Nevertheless, the abundance of the TPR-region domain containing protein was decreased specifically in Tb+ fish.

### 3.4 Protein-protein interaction networks of caudal fin proteins

**Figure 4:**
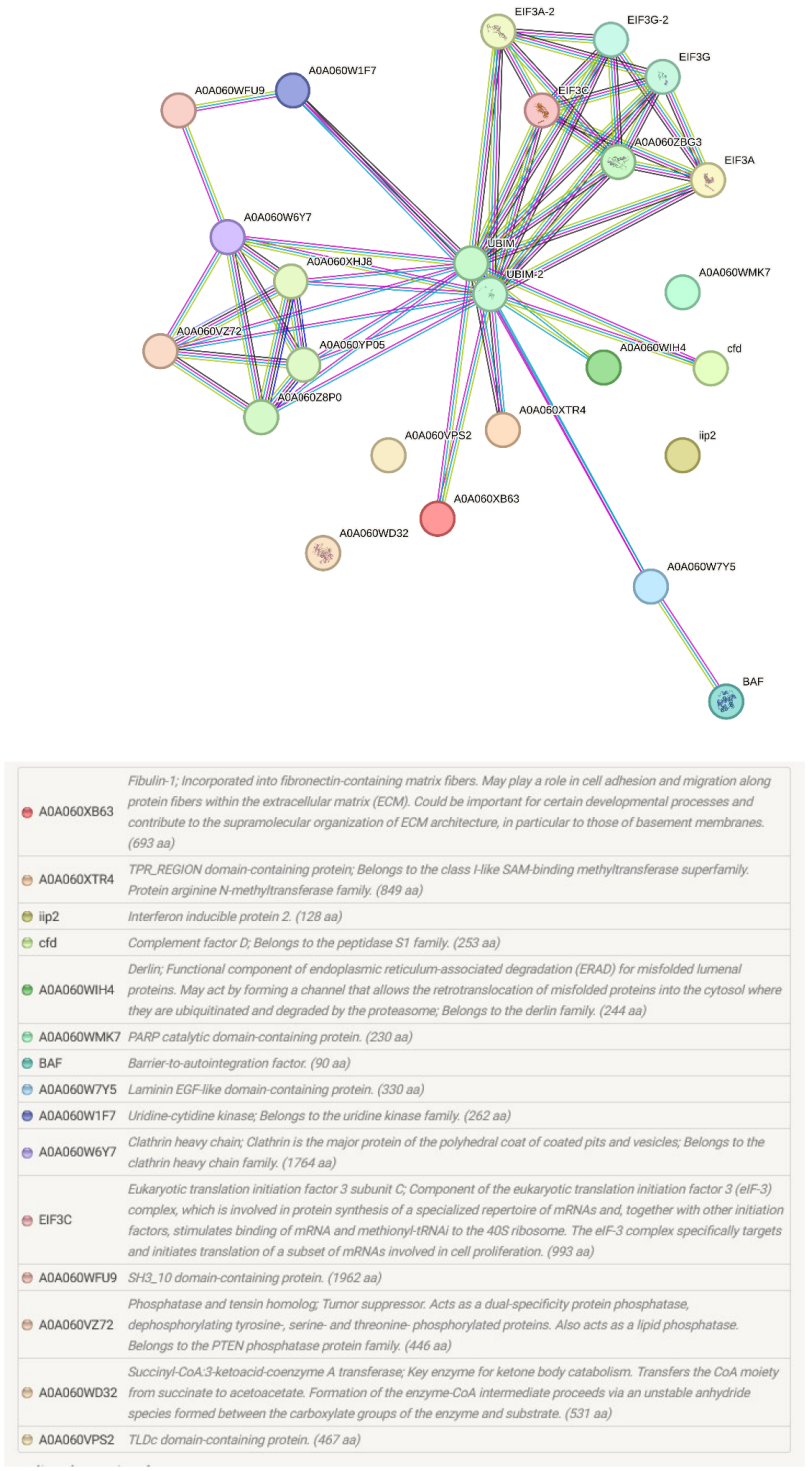
String protein–protein interaction network of the differentially regulated caudal fin proteins. In this network, nodes are proteins, lines represent the predicted functional associations, and the number of lines represents the strength of predicted functional interactions between proteins. Sixteen proteins were involved in the protein–protein interaction network.

The pathways and biological processes assigned by the KEGG orthology provided important functional information about the proteomic changes at the portals of entry of *M. cerebralis* and *T. bryosalmonae* (**Figure 4**). In our study, in addition to the identified pathways ribosome, RNA transport and lysosome, the endocytosis pathway was assigned for rainbow trout caudal fin proteins.

### 3.5 Proteins differentially regulated in gills after single infection with *T. bryosalmonae*

In the gills, 27 proteins were differentially modulated post exposure to *T. bryosalmonae* (**Table 3**). The proteomic analysis reveals different proteins involved in multiple roles such as parasite recognition, signal transduction and immune protection that were significantly upregulated after 1 day and/or 4 days. Among the upregulated proteins, two mucins, two DNA-(apurinic or apyrimidinic site) lyases, two Sodium/potassium-transporting ATPases, two C2 domain-containing proteins, B-cell receptor (CD22), and an immunoglobulin (Ig) domain containing protein are involved in host protection and immunity (**Figure 3**). In addition, many proteins involved in parasite recognition (synaptopodin, caldesmon 1b, proteases), signal transduction (protein kinase C, creatine kinase, Ryanodine receptor 1), and metabolism, repair of oxidative damage and oxidative stress (DNA-lyase, serotransferrin, splicing factor, proline-and glutamine-rich) were increased at day 1 and/or day 4 when compared with control fish or sampling time.

**Table 3:** Differentially regulated proteins in gills post *T. bryosalmonae* infection; C, Control; D, Day; Tb *T. bryosalmonae* single infection; Asterisks show significant fold changes; 100 was used for fold changes of100 or more.

Accession	Protein	TbD1/CD1	TbD4/CD1	TbD4/CD4	TbD4/TbD1
A0A060Y9M1	Serotransferrin	-100*	1.7	5.0	100*
A0A060VY25	Creatine kinase	24.8*	1.0	1.1	-23.8*
A0A8K9UM98	Mucin-5AC-like	17.3*	1.4	1.1	-12.5*
A0A8K9XRR7	Mucin-5AC	59.1*	-1.0	1.0	-62.1*
A0A8K9XIJ8	Sodium/potassium-transporting ATPase subunit alpha	66.2*	-1.1	-1.1	-75.2*
A0A8C7TYK8	Caldesmon 1b	1.9	3.3*	1.3	1.8
A0A8C7TMJ5	Splicing factor, proline-and glutamine-rich	1.2	4.1*	1.1	3.4
A0A060X5A4	Heterogeneous nuclear ribonucleoprotein M	1.4	2.1*	-1.2	1.5
A0A060VTT3	DNA-(apurinic or apyrimidinic site) lyase	-100*	1.2	-1.0	100*
A0A8C7NGB8	DNA-(apurinic or apyrimidinic site) lyase	-2.4	1.4	1.1	3.3*
A0A060XPE7	Peptidase S1 domain-containing protein	-6.5*	-2.8	-4.5	2.3
A0A8C7W9G8	DNA helicase	-100*	-1.0	-1.0	100*
A0A060Y1R0	GB1/RHD3-type G domain-containing protein	1.0	100*	100*	100*
A0A8C7SCE9	Aldo ket_red domain-containing protein	100*	1.0	1.0	-100*
A0A8C7URP6	Ryanodine receptor 1	1.4	-2.2	2.7	-3.0
A0A8K9X013	Sodium/potassium-transporting ATPase subunit beta	100*	1.0	1.0	-100*
A0A8C7NTE5	NACHT domain-containing protein	1.0	100*	1.5	100*
A0A8C7NSV2	3-hydroxyisobutyrate dehydrogenase	-100*	1.1	-1.0	100*
A0A060X058	V-set and immunoglobulin domain-containing protein 1	100*	1.0	1.0	-100*
A0A8K9UET3	C2 domain-containing protein	100*	1.0	1.0	-100*
A0A8C7S730	ATP synthase-coupling factor 6, mitochondrial	1.0	100*	1.1	100*
A0A8C7UIL6	Electron transfer flavoprotein subunit alpha	100*	1.0	1.0	-100*
A0A8C7TCX2	Synaptopodin	-1.6	1.7	1.0	2.6*
A0A060VY11	RanBD1 domain-containing protein	-100*	1.0	1.0	100*
A0A060YAQ3	C2 domain-containing protein	100*	1.0	1.0	-100*
A0A060XTK9	B-cell receptor CD22-like	100*	1.0	1.0	-100*
A0A8K9V5L9	Protein kinase C	-100*	-1.2	-1.1	100*

### 3.6 Proteins differentially regulated in gills after co-infection

At day 31 post *T. bryosalmonae* single infection and one day post co-infection, eleven proteins including seven host proteins involved in signal transduction and host immunity, were differentially modulated (**Table 4**). This included integrin beta which is involved in parasite recognition and signal transduction, which exhibited increased abundance (≥ 100) in the co-infection groups compared to the Mc group. The MHC class II antigen alpha chain showed elevated abundance (≥ 100) in the Tb+ group compared to Mc+ and the single infection groups Tb and Mc. Conversely, the abundance of the Ig-like domain-containing protein was extremely decreased (≤ 100) in the Tb+ group compared to Mc+ and the single infection groups Tb and Mc. Other proteins which showed down regulation (≤ 100) in the co-infection groups were EF-1_beta_acid domain-containing protein and the apoptotic chromatin condensation inducer 1. Fibulin 7 showed elevated abundance (≥ 100) in the Mc+ but reduction in the Tb+ group. The abundance of the all-trans-retinol 13,14-reductase was elevated (≥ 100) in the Mc+ group and Tb+ as compared to control group.

**Table 4:** Differentially regulated fish proteins in gills 31 dpe and 1 dpc; C, Control; D, Day; Mc and Mc+, *M. cerebralis* single and co-infection, Tb and Tb+, *T. brysalmonae* single and co-infection; Asterisks show significant fold changes; 100 was used for fold changes of 100 or more.

Accession	Protein	Mc+/C	Tb+/C	Mc+/Mc	Tb+/Mc	Tb+/Tb	Tb+/Mc+
A0A8C7WLA5	Integrin beta	1.0	-1.2	100*	100*	1.1	-1.3
B9VRV0	MHC class II antigen alpha chain	-100*	10.4	1.0	100*	100*	100*
A0A060YEZ9	EF-1_beta_acid domain-containing protein	-1.1	-100*	1.3	-100*	-100*	-100*
A0A060YA65	Apoptotic chromatin condensation inducer 1	1.7	-100*	1.3	-100*	-100*	-100*
A0A8C7V2W0	Ig-like domain-containing protein	-1.2	-100*	-1.0	-100*	-100*	-100*
A0A8C7S5Q0	Fibulin 7/*Oncorhynchus mykiss*	100*	1.0	1.1	-100*	1.0	-100*
A0A8C7PHI1	All-trans-retinol 13,14-reductase	100*	100*	-1.3	-1.1	1.3	1.2

**Figure 5:**
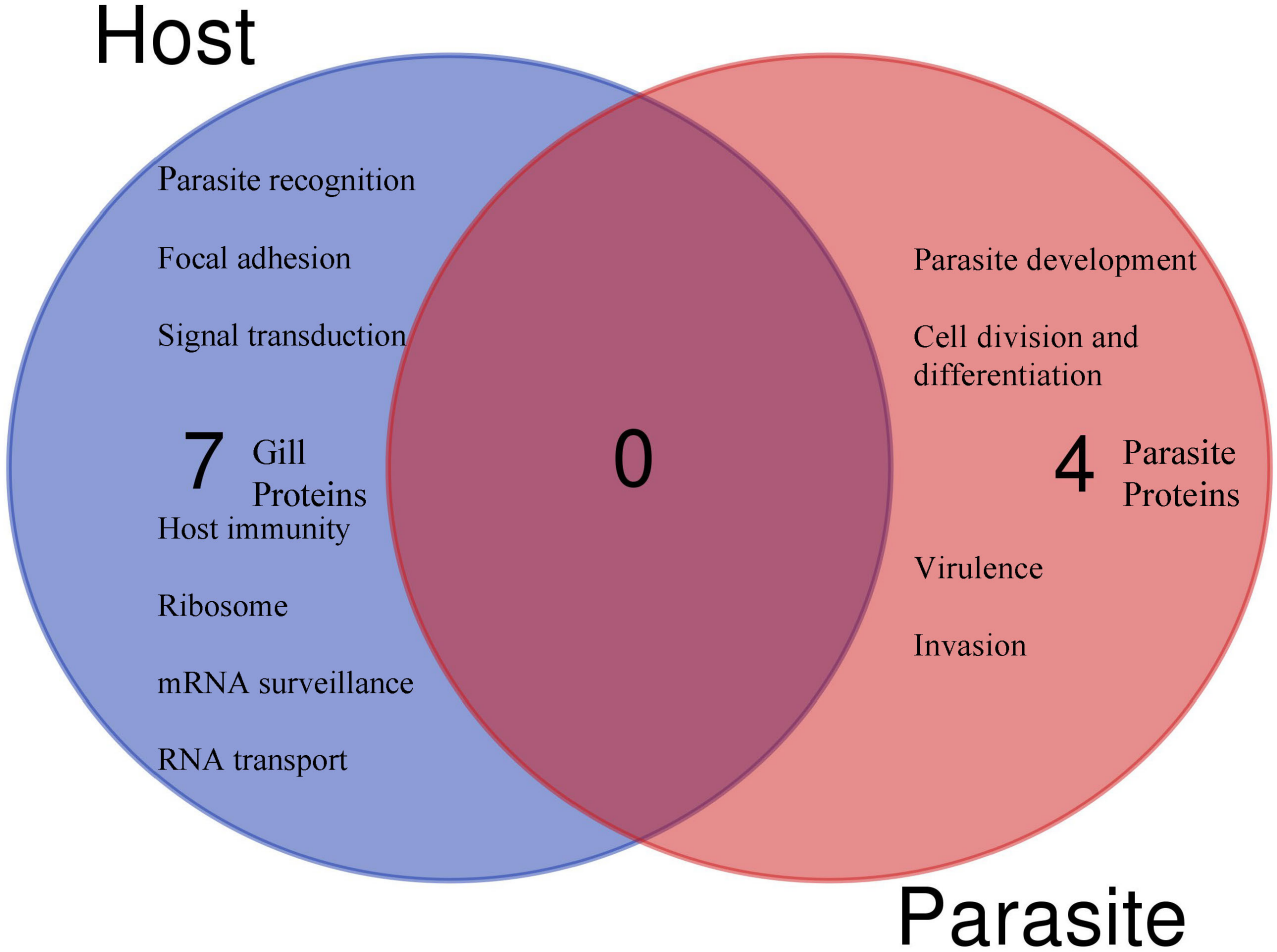
The Venn diagram shows the main molecular function and the number of unique (no shared proteins) differentially regulated parasite or host proteins identified in the gills after 30 days of single- and 1 day post co-infection, based on annotation and protein domain characterization. Venn diagram was designed using the web tool: https://bioinformatics.psb.ugent.be/webtools/Venn/.

In addition to host proteins, four myxozoan proteins were significantly upregulated (≥ 100) in gills in the co-infection Mc+ group, and included two hsp70 proteins, calmodulin and a histone (**Table 5**). The differentially regulated parasite proteins are likely involved in parasite development and virulence (**Figure 5**).

**Table 5:** Differentially regulated parasite proteins in gills 31 dpe and 1 dpc; C, Control; D, Day; Mc and Mc+, *M. cerebralis* single and co-infection, Tb and Tb+, *T. brysalmonae* single and co-infection; Asterisks show significant fold changes; 100 was used for fold changes of 100 or more.

Accession	Description	Mc+/C	Tb+/C	Mc+/Mc	Tb+/Mc	Tb+/Mc+	Tb+Tb
A0A6B2G836	Heat shock cognate 71 kDa protein	100*	1.0	-1.0	-100*	-100*	1.0
Q29W24	Heat shock protein 70	100*	1.0	1.3	-100*	-100*	1.0
A0A6B2G4Y4	Histone	100*	1.0	1.5	-100*	-100*	1.0
C1IJF2	Calmodulin	100*	1.0	-1.8	-100*	-100*	1.0

### 3.7 The protein–protein interaction network of gill proteins

**Figure 6:**
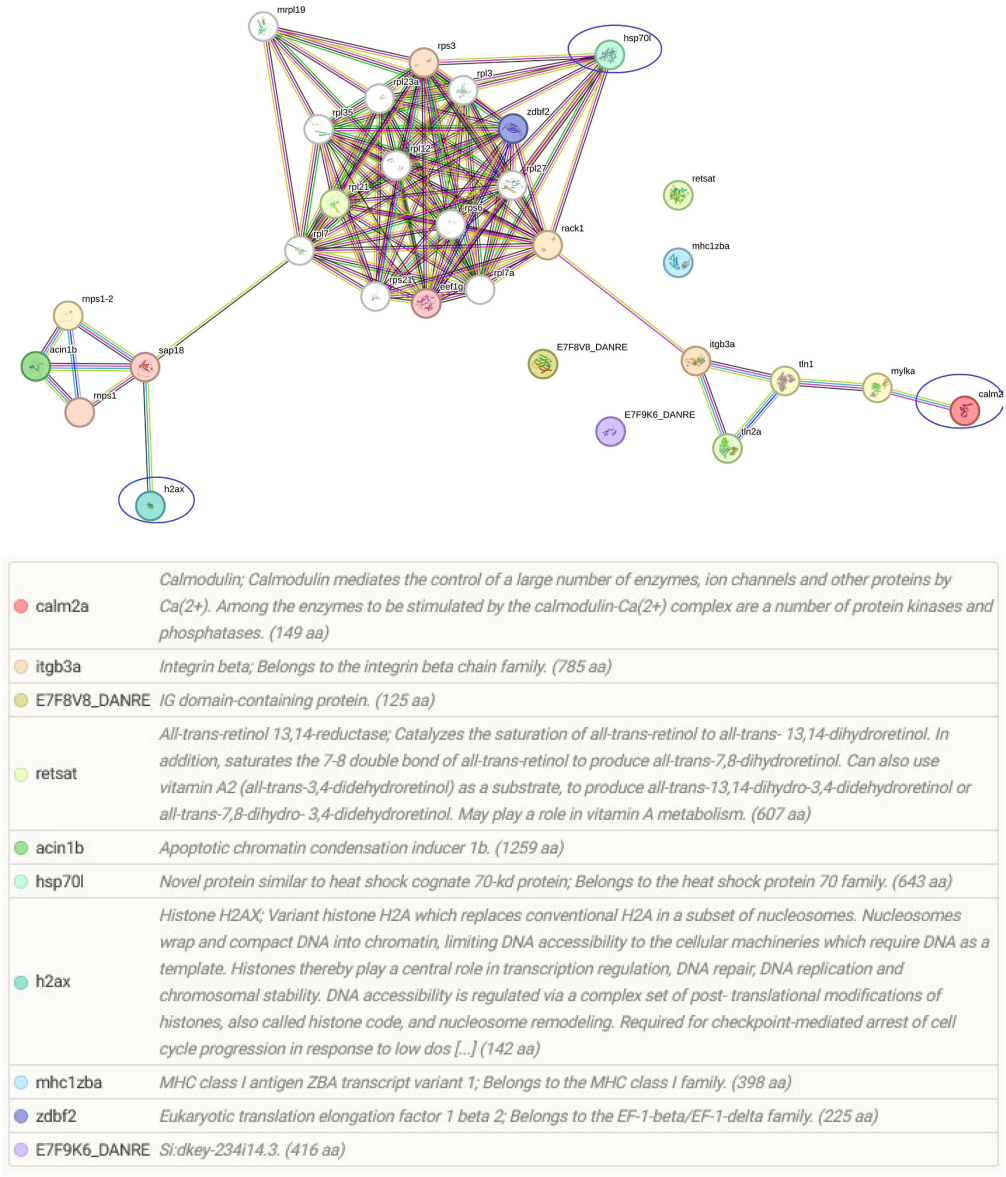
String protein–protein interaction network of the differentially regulated gill proteins. In this network, nodes are proteins, lines represent the predicted functional associations, and the number of lines represents the strength of predicted functional interactions between proteins. The figure shows that parasite proteins are associated with the host proteins.

Among the identified pathways in the gills; ribosome, mRNA surveillance pathway (to ensure quality of mRNA needed for correct translation), RNA transport (essential for gene expression), and focal adhesion are present (**Figure 5**). The protein-protein interaction network of gill proteins shows that the parasite proteins (HSP70, calmodulin and histone) are connected with the host proteins (**Figure 6**).

## 4 Discussion

Most of the studies on fish diseases focus on single infections, although co-infections are more frequent in nature. *M. cerebralis* and *T. bryosalmonae* are two freshwater myxozoan parasites with increasing economic and ecologic relevance for salmonids. Mixed infections with different myxozoans including *T. bryosalmonae* and *Myxobolus* species were reported in farmed trout in Scotland (Holzer et al., 2006). Recently, *T. bryosalmonae* co-infected with *Parvicapsula minibicornis* was detected in wild adult chum salmon in Alaska (Gorgoglione et al., 2020). The co-infection with *M. cerebralis* and *T. bryosalmonae* was established under laboratory conditions (Kotob et al., 2017; 2018), proving that these two myxozoans can co-infect the same host thus paving the way for further studies such as this one.

In this study we aimed at exploring the immunoproteomic changes induced by the two parasites at the preferred portal of entry (caudal fins for *M. cerebralis* and gills for *T. bryosalmonae*) during single and co-infection in rainbow trout. The proteomic profiles highlighted traits of host recognition and invasion as well as of the immune response of rainbow trout to co-infection with the two myxozoan parasites. The obtained results help us explain and better understand the host-parasite interaction in rainbow trout in response to *M. cerebralis* and *T. bryosalmonae* at the portals of entry. These results, discussed below, show that depending on the order of infection with the two myxozoans and the portal of entry, the mucosal responses varied considerably, indicating differential priming of mucosal tissue responses by the two species.

### 4.1 Myxobolus cerebralis and Tetracapsuloides bryosalmonae parasite burden

The highest load of *M. cerebralis* was observed on 4 dpe, indicating the suppressive effect of *M. cerebralis*. During the course of the single infection with *M. cerebralis*, the spores successfully parasitize, multiply in the caudal fin and suppress the host immune responses. The parasite load decreases considerably on 31 dpe (1 dpc), as the parasites leave the infection site to reach the target organ (head cartilage) (Saleh et al., 2019a). The increased *M. cerebralis* load in the caudal fin indicates that the fish are not able to maintain proper tissue hemostasis, immunity or limit parasite burden during WD as previously reported (Severin and El-Matbouli, 2007; Severin et al., 2010; Baerwald, 2013; Kotob et al., 2018; Saleh et al., 2020a; 2020b). On the other hand, the *T. bryosalmonae* burden decreased over time showing and the lowest parasite count was observed in the Tb+ group on 1 dpc. The reduced load in the gills implies that the fish are aimed at maintaining proper tissue hemostasis, immunity and limit parasite burden (Gorgoglione et al., 2013; Kotob et al., 2018; Bailey et al., 2020).

### 4.2 Caudal fin proteome modulation after single infection

Ly6/uPAR domain-containing protein was one of the significantly increased proteins in caudal fin in the Mc infected fish. It is a known biomarker of inflammation, present in most metazoans and plays important roles in different biological processes including host immunity, cellular adhesion, and cell signalling (Guo et al., 2015). The increased level of this protein at day 4 in the Mc infected fish indicates an involvement in the immune response to *M. cerebralis* and in signal transduction during WD. Indeed, Ly6/uPAR is known to be involved in disease pathology in severe malaria, viral infection and virus-host interactions, and can enhance virus infection by targeting viral entry (Plewes et al, 2014; Mar et al., 2018; Yu et al., 2019). Similarly, Ly6/uPAR might be involved in *M. cerebralis* infection and fish-myxozoan interactions. During WD, uncontrolled inflammation augments tissue damage triggered by *M. cerebralis*, giving a possible means for Ly6/uPAR to enhance parasite entry and increase parasitic burden (Saleh et al., 2019a; 2020a).

Another interesting finding was in relation to the SH3 domain containing glutathione-*S*-transferase (GST)-fusion protein that strongly inhibits the formation of endocytic clathrin-coated vesicle (CCV) in permeabilized cells (Simpson et al., 1999). Clathrin-mediated endocytosis (CME) is a major route of entry into eukaryotic cells. In fact, exceptionally fast CME turnover aids *Trypanosoma brucei* evasion of the host immune system (Manna et al., 2015). At day 4 post exposure to *M. cerebralis*, the reduced abundance of the SH3-domain-containing protein points to a non-restricted increase of endocytic CCV formation. This likely occurs as the abundance of the clathrin heavy chain protein increases, as seen at day 4 in the Mc infected fish. Taken together, this seems to be part of the parasite invasion strategy through endocytic CCV formation. Apparently, *M. cerebralis* uses the CME to facilitate its entry into the host and evade the fish immune system. The endocytosis pathway was also reported to have an important role for *T. bryosalmonae* nutrition acquisition and virulence (Morris and Adams, 2008), recently confirmed by transcriptomic analysis of PKD infected kidney tissue (Ahmad et al., 2021; Faber et al., 2021). After exposure to *M. cerebralis*, within minutes the sporoplasm (contains 64 internal cells) migrates to deeper layers of the epidermis, then within hours of initial infection, aggregates and single cells from the sporoplasm begin mitotic replication alternating between inter and intracellular locations, which is a critical phase in the early stages of *M. cerebralis* infection, invasion and replication in trout (El-Matbouli et al., 1995; Baerwald et al., 2008). This pathway is used as an evasion strategy by infectious hematopoietic necrosis virus (IHNV) in rainbow trout (Liu et al., 2011). The CME pathway is also used by *Piscirickettsia salmonis* for host entry (Ramírez et al., 2015), which suggests that the CME mechanisms are involved in the transport of large particles and pathogens in fish. Hence, this pathway might be also used by *M. cerebralis* as an evasion strategy for speedy parasite uptake. However, functional assays are needed to prove whether *M. cerebralis* uses this pathway as an evasion strategy.

Among the differentially regulated proteins identified in common carp skin mucus after exposure to the deleterious parasite *Ichthyophthirius multifiliis*, three interferon (IFN)-induced GTP-binding Mx-like proteins were upregulated (Saleh et al., 2019b). These proteins are implicated in complement activation, which leads to opsonization, leucocyte stimulation, and direct pathogen killing (Das et al., 2019). These proteins are released to prevent pathogen attack and regulate cellular activities such as endocytosis and trafficking of nucleoproteins into the nucleus (Haller and Kochs, 2002). They also play an important role in parasite recognition and signal transduction (Timmerhaus et al., 2011; Dawar et al., 2016; Saleh et al., 2019b). In fish, the Mx gene(s) is typically induced by Type-I IFN, the main cytokine mediating the innate immune function (Das et al., 2019). However, some salmonid Mx genes induced by IFN-γ (Type-II IFN) have been reported recently (Robertsen et al., 2019; Wang et al., 2019). Since the expression of trout IFN-γ is known to be increased after exposure to *M. cerebralis* (Baerwald et al., 2008; Saleh et al., 2020b), this suggests a link to the current findings where the two IFN-induced GTP-binding Mx proteins may have been activated by IFN-γ-mediated innate immunity. The abundance of the two GTP-binding Mx proteins was differentially increased for one protein at 4 dpe and for the second at 1 and 4 dpe. The results suggest their involvement in diverse activities including parasite recognition, signal transduction and host protection during infection by *M. cerebralis.*

Fibulin-1 and laminin were also highly increased post-infection. Fibulin-1 has a key role in remodelling the extracellular matrix (ECM) of the fin and is a main constituent of the ECM. It can mediate cell signal transduction by binding to ECM proteins such as laminin and fibronectin (Martin et al., 1988). Zebrafish fibulin-1 was also found to be expressed in fins as well as numerous other tissues (Feitosa et al., 2012). The increased abundance of fibulin-1 in *M. cerebralis* infected rainbow trout (as well as in unexposed fish) points to its involvement in fin development as well as in parasite recognition and signal transduction.

The EGF-like domains in laminin and in other ECM proteins, are indicators for cellular growth and differentiation. These domains stimulate adjacent cells in a specific and precise manner (Engel, 1989). The upregulation of laminin in a similar manner as fibulin-1 points to an association of these two proteins in the rainbow trout ECM and their involvement in cellular development, growth promotion, tissue repair and signal transduction.

PARP was also increased in single infected caudal fins, although not to the same extent as fibulin-1 and laminin. PARPs are involved in chromatin regulation, transcription, RNA biology, and DNA repair. PARP, as a prominent marker of apoptosis was upregulated in rainbow trout, revealing apoptosis induction under endoplasmic reticulum stress (Séité et al., 2019). PARP acts on both host and viral proteins through binding to deltex E3 ubiquitin ligase 3L (DTX3L) to mediate efficient immune responses to pathogens (Gupte et al., 2017). The catalytically inactive PARP-9 associates with DTX3L to recruit immune responses to viral pathogens (Zhang et al., 2015). This binding stimulates E3 ubiquitin ligase activity and promotes IFN-stimulated gene expression. During WD, ubiquitin like protein 1 was upregulated by more than 100-fold and IFN regulating factor 1 (IRF 1) was upregulated by more than 15 fold after exposure to *M. cerebralis* (Baerwald et al., 2008). In the current study, the rainbow trout 40S protein S30, which belongs to the ribosomal ubiquitin like protein eS30 fusion protein (UBIM) was predicted to be a functional partner and is the central node of the protein–protein interaction network of caudal fin proteins (**Figure 4**). IFN activates its downstream transcription factor STAT1 and successive binding of STAT1 to genomic loci to stimulate proinflammatory gene expression. In WD, several studies described IFN as a common link in the response to *M. cerebralis* (Baerwald et al., 2008; Saleh et al., 2020b). In fact, the expression of many proinflammatory genes was upregulated in response to *M. cerebralis* (Rucker and El-Matbouli, 2007; Severin and El-Matbouli, 2007, Severin et al., 2010; Baerwald, 2013; Saleh et al., 2020a; 2020b). The increased expression of STAT1 and several proinflammatory molecules including IFNγ, IRF 1, iNOS, Nramp α, Nramp β, IL-1β, STAT3 and IL-17 during WD suggests that PARP is also involved in the activation of the fish immune response against *M. cerebralis*.

In this study, the slingshot protein of rainbow trout was apparently increased in the Mc group at 1 and 4 dpe, presumably to retain the cytoskeleton structure and cope with tissue disruption due to parasite proliferation. In *Drosophila*, the slingshot protein family was shown to play a pivotal role in actin dynamics by reactivating cofilin *in vivo* (Niwa et al., 2002). Derlin is a component in the endoplasmic reticulum degradation (ERAD) pathway and is required for the dislocation and degradation of certain misfolded proteins from the ER to the cytosol (Lilley and Ploegh, 2004). The increased abundance of derlin heavy chain after exposure to *M. cerebralis* indicates a possible involvement in degradation of misfolded proteins due to stress caused by parasite proliferation during WD. In zebrafish, the TLDc domain-containing protein (oxidative resistance gene 1) is crucial for defence against oxidative stress (Xu et al., 2020). TLDc domain-containing proteins were shown recently to be modulated in the fish skin proteome after parasitic infection (Saleh et al., 2019b). The increased abundance of the TLDc domain-containing protein here indicates that the host attempts to reduce oxidative stress caused by *M. cerebralis* as a means to protect rainbow trout against infection.

Carp cytidine monophosphate kinase 2 (CMPK2) is upregulated by bacterial challenge and has a protective effect on the gut barrier, suggesting that teleost CMPK2 is involved in innate immune responses against bacterial and viral infections (Liu et al., 2019; Feng et al., 2021). CMPK2 was also upregulated in Atlantic salmon in response to infection with the monogenean parasite *Gyrodactylus salaris* and after injection with the immunostimulants LPS and Poly (I:C) (Collins et al., 2007). So far these are the only sources available that signify its potential antiparasitic and antibacterial function in fish. The increased abundance of this protein in *M. cerebralis* infected fish indicates that CMPK2 might have a similar protective function in rainbow trout immunity against parasites as in Atlantic salmon. The succinyl-CoA:3-ketoacid-coenzyme A transferase (SCOT) catalyzes the conversion of CoA from succinyl-CoA to acetoacetate to produce acetoacetyl-CoA and succinate. Acetoacetyl-CoA is further converted into acetyl-CoA, which passes in the citric acid cycle to either deliver energy or be stored as a fatty acid (Zhang et al., 2013). The increased SCOT level at day 4 in this study indicates that parasite propagation [highest parasite load was observed at day 4 post exposure to *M. cerebralis* (**Figure 2A**)] likely influences metabolism in the fish host.

The barrier to autointegration factor (BAF) is known to be used by viruses to improve infectivity and protein biosynthesis. In fact, BAF expression is increased in Atlantic salmon following exposure to infectious salmon anemia virus (Workenhe et al., 2009). In addition, Atlantic salmon BAF is upregulated in response to IHNV, which is thought to facilitate viral protein transcription within the host DNA (Miller et al., 2007). So far these are the only sources available that signify its potential antimicrobial function in fish. BAF is likely involved in the fish response to myxozoan parasites and the increased abundance at day 4 suggests that *M. cerebralis* uses BAF to increase infectivity and propagation in rainbow trout similar to IHNV in Atlantic salmon. The abundance of the eukaryotic translation initiation factor 2B (EIF2B) is involved in chromatin organization and represses gene expression in the early response of Atlantic salmon to infection (Krasnov et al., 2012). This protein was downregulated in rainbow trout following infection with *Aeromonas salmonicida*, which likely decreases translation generally but at the same time can increase the translation of stress response mRNAs (Causey et al., 2018). The increased abundance of EIF2B at day1 and day 4 might suggest its involvement in the rainbow trout early immune response against *M. cerebralis*.

### 4.3 Caudal fin proteome modulation after coinfection

After coinfection, the relatively low number of the differentially regulated protein identified was actually anticipated and can be attributed to the waning of the initial response of the single infection as the parasites leave the skin and gills after 4 day and subsequently most of the preliminary local responses are no longer present at day 31.

Complement factor H is a multidomain, multifunctional protein, which can bind to a range of ligands and protect host cells against damage mediated by disturbance of complement system (Wei et al., 2022). This inhibitory regulator of the alternative pathway of the complement system prevents the synthesis of C3 convertase to suppress complement activation (Du Clos and Mold, 2008). In Nile tilapia, the complement factor H protein has a robust binding capacity to pathogenic bacteria and is involved in the maintenance of homeostasis within the host and pathogen (Wei et al., 2022). The increased abundance in the co-infection groups indicates that the complement factor H protein likely contributes to tissue haemostasis and the immune response of rainbow trout against myxozoan co-infections. On the other hand, the down regulation of serine/threonine protein phosphatase could be a part of the immune evasion strategy of *T. bryosalmonae* to suppress host immunity and support subsequent co-infections. TPR-region domain containing protein, which controls protein organization and haemostasis was differentially modulated in the co-infections group. Indeed, during *T. bryosalmonae* infection a general immunosuppression of the fish host is observed as a parasitic strategy to evade the immune system like that of protozoan parasites (Bailey et al., 2020).The abundance of the TPR-region domain containing protein was extremely reduced in the Tb+ group, although the parasite load was decreased compared to the other groups, signifying that the primary suppressive action after *T. bryosalmonae* exposure was endorsed at 31 dpe (1 dpc) apparently to limit inflammatory responses, tissue destruction and subsequent invasion with *M. cerebralis* (seen by the decreased parasitic load) and to maintain tissue haemostasis properly.

### 4.4 Gill proteome modulation after single infection

Several proteins were differentially regulated in the gills after exposure to *T. bryosalmonae*. The host protection and immunity proteins serotransferrin, mucin, B-cell receptor (CD22), and Ig domain containing protein were increased at day 1 and/or day 4. The highest load of *T. bryosalmonae* observed in the Tb group at day 1, was decreased at day 4 and the lowest parasite count was observed in the Tb+ group at day 31 (day 1 after co-infection) (**Figure 2B**).

Indeed, previous studies have shown that *M. cerebralis* and *T. bryosalmonae* can induce B- and T-cell associated molecules (Saleh et al., 2019a; Bailey et al., 2020). Moreover, several proteins including synaptopodin, caldesmon 1b and proteases likely involved in parasite recognition were differentially modulated. Other proteins such as protein kinase C, creatine kinase and ryanodine receptor 1 may have roles in signal transduction. Furthermore, proteins involved in transport, metabolism, repair of oxidative damage and oxidative stress including sodium/potassium-transporting ATPase subunit alpha and beta, DNA-lyase and splicing factor (proline-and glutamine-rich) were differentially abundant at day 1 and/or day 4. Indeed, multiple proteins with similar functions were previously reported modulated in response to parasitie infections in fish (Saleh et al., 2019b). In the current study, the abundance of the proteins involved in host immunity and protection was mostly induced at day 1 compared to control fish, whereas they were reduced at 4 compared to 1 dpe to *T. bryosalmonae*. On the other hand, the abundance of the proteins involved in metabolism and repair of oxidative damage was increased at day 1 compared to control group and reduced at 4 compared to 1 dpe, indicating that rainbow trout is able to maintain proper haemostasis, limit inflammatory reactions and provide adequate immune responses against *T. bryosalmonae*.

### 4.5 Gill proteome modulation after co-infection

Integrin was identified among the differentially regulated proteins after co-infection in the gills. In mammals, it is involved in cellular processes such as focal adhesion, signal transduction, interactions, cell migration, growth and differentiation, cell-cell and cell-pathogens (Yamada and Danen, 2000). Integrins are cell receptors that can bind the protein components of viral proteins, and play crucial roles in fish immune responses to viral infection by inducing Type I IFN (Xiong et al., 2022). This protein had highest abundance in the co-infection groups compared to the *M. cerebralis* single infection fish, which may indicate that *T. bryosalmonae* induces rainbow trout integrin to limit a secondary infection from other myxozoans (in this case to limit *M. cerebralis*). A further protein showing elevated abundance in the co-infection groups, as compared to control fish was the all-trans-retinol 13,14-reductase, also known as retinol saturase. In mammals, it is implicated in glucose and lipid metabolism, macrophage function, vision, and the generation of reactive oxygen species (Weber et al., 2020). In the current study, increased abundance in Mc+ and Tb+ fish suggests its involvement in the host stress response to myxozoan co-infections.

Focal adhesion was identified among multiple pathways by the protein-protein interaction network analysis of gill proteins in this study. The focal adhesion pathway acts as a signal sensor to mediate signal transduction and the immune response to stimuli. Indeed, a previous study has shown that parasitic infection (*I. multifiliis*) stimulated the focal adhesion pathways in rainbow trout (Roh et al., 2021).

Lastly, the upregulation of B-cell markers (MHC II, B-cell receptor CD22, Ig-domain containing protein) was also seen in the gills, mainly in the Tb single and/or co-infection groups. This may link to previous studies that have shown that *M. cerebralis* and *T. bryosalmonae* can induce B- and T-cells (CD8+ and CD4+ cells) (Saleh et al., 2019a; Bailey et al., 2020), potentially here as part of the local mucosal response at this site. Indeed, the importance of IgT^+^ B cell responses in trout gills infected with other types of parasites (*I. multifiliis*) is well known (Xu et al., 2016).

### 4.6 Parasite proteins

In addition to the modulated host proteins, four myxozoan proteins were also found to be significantly increased in Mc+ fish gills. These included two hsp70, calmodulin and histone. These myxozoan proteins apparently have a role in parasite virulence and growth. Specifically, hsp70 has a role in cell structure, cell regulation, protein assembly, folding, and translocation during parasitic infections in fish (Abernathy et al., 2011; Saleh et al., 2021). In a recent transcriptome study, Hsp60, 70, and 90 *T. bryosalmonae* homologues were found to be abundantly expressed in bryozoa and fish suggesting potential contribution to parasite survival, temperature-driven development and host invasion (Faber et al., 2021; Shivam et al., 2023). Interestingly, as a vaccine adjuvant, hsp70 gives high protection against the fish parasite *Cryptocaryon irritans* in orange spotted grouper (Josepriya et al., 2015). Hence, it is worth to functionally investigate whether and how myxozoan hsp70 could exert such protection in fish. The *M. cerebralis* histone was found connected to host proteins in the protein–protein interaction network. This protein is likely essential for accelerated cell division and myxozoan stage differentiation, as seen in a previous study of *Myxobolus bejeranoi* where differential expression of histones were found to be associated with parasitic invasion (Maor-Landaw et al., 2023). Calmodulin was also involved in the gill protein-protein interaction network, and is likely a key virulence factor that contributes to parasite motility and invasion of myxozoan parasites, as reported for other parasites (Long et al., 2017).

## 5 Conclusion

Proteome profiling of the rainbow trout portals of entry for *M. cerebralis* and *T. bryosalmonae* single and co-infections revealed novel insights into myxozoan elicited protein changes in the fish host. *M. cerebralis* and *T. bryosalmonae* modulated host defence responses and different host proteins were produced during each co-infection. The differentially regulated proteins were associated with signal transduction, host immunity, metabolism and tissue repair as well as host parasite interaction. In addition, several myxozoan proteins (2 members of hsp70, histone and calmodulin) involved in virulence and invasion were found to be differentially regulated in the gills of the Mc+ group (fish initially infected with Mc then 31 days later co-infected with Tb). This suggests that host immune suppression by *M. cerebralis* could influence a subsequent co-infection by *T. bryosalmonae*. Thus, these results enhance our knowledge of the virulence strategies of the two parasites, especially in relation to the elevated abundance of proteins associated with virulence and invasion.

It seems clear that *M. cerebralis* modulates the rainbow trout immune response in a way that likely synergistically supports a subsequent co-infection with *T. bryosalmonae*. On the other hand, *T. bryosalmonae* activates the host immunity (especially B cell-mediated immunity, as evidence by induction of the B-cell markers MHC II, B-cell receptor CD22 and Ig-like domain containing protein) and may antagonistically alter the outcome of a subsequent co-infection with *M. cerebralis*. The biological processes, pathways and molecular functions of the differentially regulated proteins were also analysed. Multiple proteins were involved in host parasite interactions and immune response of rainbow trout to co-infection with myxozoan parasites give better understanding of these host-parasite interactions. Possible invasion/evasion strategies associated with clatherin-mediated endocytosis, as well as induction of key parasite virulence proteins (hsp70, histone and calmodulin) during myxozoan infections at the portals of entry were highlighted. However, functional assays are needed to investigate the precise role of these proteins in host invasion and whether *M. cerebralis* uses the clathrin-mediated endocytosis pathway to evade the host immune response. Future proteomic studies of target organs (head cartilage and kidney) as well as analysis of the transcriptomic changes at the portals of entry and target organs will help elucidate further how myxozoans modulate and influence the outcome of rainbow trout co-infection.

## Data availability statement

The data presented in the study are deposited in the ProteomeXchange Consortium via the PRIDE partner repository, accession number PXD050412.

## Ethics statement

The animal study was approved by Ethics Committee of Vienna University of Veterinary Medicine (BMBWF-V/3b-2021-0.240.416). The study was conducted in accordance with the local legislation and institutional requirements.

## Author contributions

MS: Conceptualization, Funding acquisition, Investigation, Methodology, Project administration, Validation, Writing – original draft, Writing – review & editing. KH: Data curation, Formal analysis, Investigation, Methodology, Software, Validation, Writing – review & editing. SS: Data curation, Formal analysis, Methodology, Validation, Writing – review & editing. ER-F: Data curation, Formal analysis, Software, Validation, Writing – review & editing. JB: Formal analysis, Validation, Writing – review & editing. AH: Resources, Writing – review & editing. CJS: Conceptualization, Writing – review & editing. ME: Conceptualization, Writing – review & editing.
